# Bioinformatics driven in gene targeting platform for gold anticancer strategy delivery

**DOI:** 10.1016/j.mtbio.2025.102438

**Published:** 2025-10-18

**Authors:** Can Jiang, Haixuan Wen, Jiabin Chen, Na Han, Xin Sun, Yuzhu Zhang, Yongbin Hu, Guang Shu, Gang Yin, Maonan Wang

**Affiliations:** Department of Pathology, Xiangya Hospital, Xiangya School of Basic Medical Sciences, Central South University, Changsha, China

**Keywords:** Triple-negative breast cancer, SDC1, Single-cell sequencing, Nucleic acid delivery system

## Abstract

Owing to its high degree of malignancy and poor survival outcomes, triple-negative breast cancer (TNBC) is considered the most invasive subtype of breast cancer. In the realm of TNBC treatment, clinical practice continues to be predominantly characterized by the utilization of chemotherapy regimens. The development of anticancer therapies that are specifically targeted and precise in their action remains a significant challenge within this therapeutic domain. This study aims to discover new target genes and develop nucleic acid delivery systems. In this study, we identified the differentially expressed gene SDC1, which exhibited high levels of expression in TNBC and correlates with poorer overall survival trends through a comprehensive gene chip data screening analysis. The results of our analysis suggest a positive correlation between increased SDC1 expression levels and etoposide drug resistance in cases of TNBC. For mechanistic insights, scRNA-seq was employed to map SDC1-dependent alterations in the tumor microenvironment (TME) immune architecture. In view of this, the present study successfully constructed an *in situ* self-reactive gold nanocluster SDC1 shRNA-targeted nucleic acid delivery system in tumor cells by taking advantage of the reductive microenvironment at the tumor site, which significantly inhibited TNBC angiogenesis. This study elucidated the molecular mechanism by which SDC1 promotes tumor progression through multidimensional modulation of the TNBC microenvironment. It also proposed a bioinformatics-driven gene-targeting integrated platform for the rational design and delivery of gold nanocluster-based anticancer strategies.

## Graphical abstract

**Figure:**
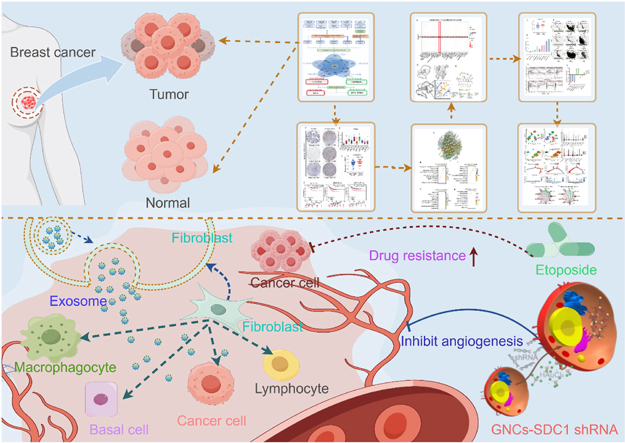

## 1 Introduction

As stated in the Global Cancer Statistics Report 2024, breast cancer holds the distinction of the highest incidence (2,309,000 cases, constituting 23.8 % of the global cancer burden) and mortality (662,000 deaths, representing 15.4 % of all cancer-related fatalities) among female patients worldwide [1]. Per molecular classification, TNBC denotes ER-/PR-/HER2-breast cancer exhibiting peak malignancy potential and minimal survival expectancy among mammary carcinomas [[2], [3], [4], [5]]. Hence, improving the long-term survival and prognosis of triple-negative breast cancer patients is an urgent priority. Currently, the primary treatment modalities for TNBC are surgical interventions and chemotherapy with adjuvant radiotherapy [6,7]. As research into triple-negative breast cancer has progressed, endocrine therapy, targeted therapy, and immunotherapy have enhanced the prognosis of patients with this condition to a certain extent. However, the high malignancy and frequent development of treatment resistance in triple-negative breast cancer mean that most patients do not benefit from various therapies [[8], [9], [10], [11], [12]]. Furthermore, the triple-negative breast cancer tumor microenvironment (TME) exerts significant influence on tumor progression, metastasis, and treatment resistance. The TME is a complex ecosystem comprising a heterogeneous mixture of multiple cell types, signaling molecules, and physicochemical factor interactions [13,14]. The dearth of effective therapeutic options and the poor prognosis associated with triple-negative breast cancer present a significant challenge in the effective management of this highly lethal form of cancer. Advances in drug delivery technology are transforming the way pharmaceutical treatments are managed, shifting the focus from “extensive management” to “intelligent, precision-controlled regulation”. This shift is key not only to reducing side effects, but also to enhancing therapeutic efficacy [15]. Consequently, there exists a pressing need to identify novel targets and develop delivery systems around them to improve the prognosis of patients with TNBC.

Presently, an extensive array of tumor data from myriad cancer databases is at our disposal for research purposes. Notably, the Cancer Genome Atlas (TCGA) project houses a substantial repository of clinical data, alongside mRNA and microRNA expression profiles, methylation data, and additional information relevant to diverse oncological conditions, including their respective subtypes [16]. Serving as a repository, the Gene Expression Omnibus(GEO)database provides high-throughput gene expression data submitted by research institutions around the world including a variety of publicly available data sets such as tumor and non-tumor microarrays, differential analysis, and molecular validation, among others [17]. In this study, we initially obtained breast cancer-related data from the GEO databases (GSE42568, GSE65194, GSE45827, GSE38959) and the TCGA database. Ensuing investigations focused on the differentially expressed genes revealed by comparative analysis of breast cancer versus normal breast tissues. Through a series of analyses and screenings, SDC1 was ultimately identified that exhibited a positive association with the poor prognosis of TNBC and was found to be highly expressed in patients with TNBC. SDC1, also known as Syndecan-1, is a transmembrane glycoprotein that is encoded by the SDC1 gene. It is a member of the heparan sulfate acetate proteoglycan (HSPG) family [18]. Research on TNBC has indicated that elevated SDC1 expression activates the c-SRC/FAK signaling pathway, thereby promoting migration. However, the specific pathogenic mechanisms of SDC1, including chemoresistance, single-cell analysis, and the design of delivery systems, remain to be elucidated [[19], [20], [21]]. Consequently, we elected to designate SDC1 as the primary subject of our subsequent study.

**Scheme 1:**
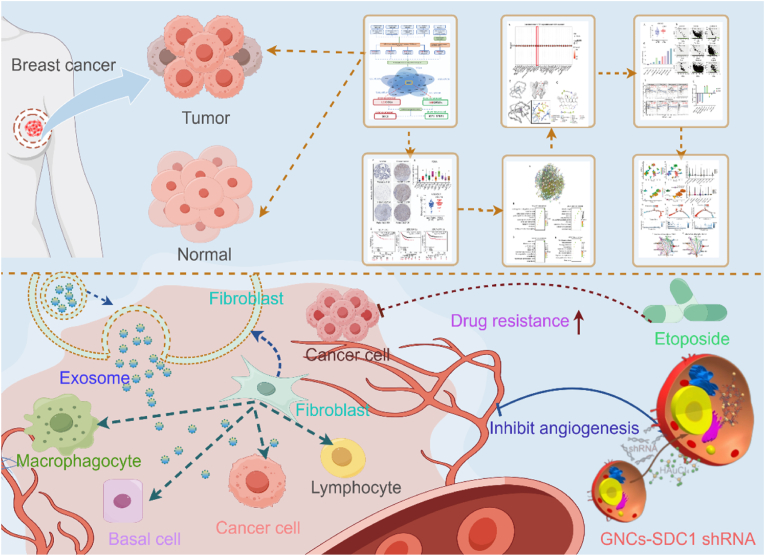
*In situ* self-reactive gold nanoclusters enable targeted delivery of SDC1 shRNA and inhibit triple-negative breast cancer angiogenesis by suppressing etoposide resistance and tumor microenvironment reprogramming.

In order to investigate the mechanism of SDC1 in TNBC, we performed a functional enrichment analysis. GO and KEGG enrichment analysis revealed that SDC1 may generate chemoresistance by modulating the PI3K-Akt signaling pathway and Rap1 signaling pathway. Furthermore, a drug sensitivity analysis demonstrated that higher levels of expression of SDC1 was substantially and positively associated with resistance to etoposide. In addition, SDC1 and etoposide may have a direct interaction (binding energy −7.6 kcal/mol); notably, an analysis of the GEO database revealed that SDC1 exhibited a high expression feature that was age-dependent in elderly patients with TNBC. Moreover, a marked positive correlation was detected between SDC1 expression and cellular senescence in TME, suggesting that SDC1 may serve as a potential tumor senescence-related marker. For deeper mechanistic insights into SDC1's involvement in conferring treatment resistance and senescence, an analysis of the immune cell populations in the TME was conducted. This analysis revealed that SDC1 may play an immunomodulatory role by affecting CD8^+^ T cell and macrophage infiltration. Single-cell sequencing analysis revealed that SDC1 was specifically highly expressed in fibroblasts in the TME Combined with our functional enrichment analysis results, the SDC1 may exist exosome, Consequently, exosomal paracrine secretion potentially enables SDC1 to orchestrate intercellular communication between fibroblasts and neighboring cells within the TME. Pseudo-temporal trajectory analysis further confirmed that its expression dynamics were mechanistically linked to the differentiation process of cancer-associated fibroblasts (CAFs), and SDC1 may be involved in the construction of a pro-tumor microenvironment through an exosome-mediated paracrine mechanism (Scheme 1).

Finally, it is noteworthy that a self-initiated *in situ* fluorescent GNCs-RNA complex nucleic acid delivery system was designed, taking advantage of the highly reducing microenvironment at the tumor site. This system enabled the innovative *in situ* delivery of SDC1 shRNA. The successful construction of this delivery system was detected by characterization methods such as TEM, HRTEM, Raman spectroscopy, and FTIR. Compared with commercial transfection reagents-Neofect, this system not only had higher nucleic acid delivery efficiency, but also inhibited tumor angiogenesis while achieving better fluorescence-targeted imaging. Overall, a comprehensive analysis of single-cell data and genome-wide biosignature analysis revealed that SDC1 exhibited high levels of expression in TNBC and demonstrated a significant positive correlation with patients' worse prognosis. It is hypothesized that SDC1 plays a role in the construction of a pro-tumorigenic microenvironment, influencing immune cell infiltration, and promoting angiogenesis through an exosome-mediated paracrine mechanism. Concurrently, a personalized *in situ* self-assembly nucleic acid delivery system was designed, ensuring both safety and reliability. In light of these findings, we hypothesize that SDC1 will emerging as a prioritized target for TNBC therapy. The development of a delivery system targeting SDC1 offers a significant and promising therapeutic approach for the effective clinical management of TNBC.

## 2 Materials and methods

### 2.1 Materials

Medium was purchased from Yuanpei (Wuhan, China), serum was purchased from Pronosay (Wuhan, China), and shRNA was synthesized by Miaoling Biosynthesis (Wuhan, China) using the sequence of siRNA-1. Chloroauric acid was obtained from Maclean's (Shanghai, China).

### 2.2 Methods

#### 2.2.1 Genetic difference analysis

UALCAN (https://ualcan.path.uab.edu/) is an online platform for data analysis based on the TCGA database, including transcriptional level difference analysis, survival analysis, correlation analysis, gene and protein level difference analysis, miRNA and lncRNA analysis, etc. Meanwhile, the database can be utilized to realize the analysis results of the TCGA cancer genomics data. The analysis results of the database can be visualized. Utilizing this database, we examined SDC1 expression levels in TNBC. R (version 4.4.1) is a free and open source software. We used R to compute a large amount of data from TCGA database and GEO database and screened out the eligible differentially expressed genes with |logFC|≥1 and *P* ≤ 0.05 as the filtering condition. In addition, we used GraphPad Prism 10 (version 10.1.2) software to analyze and visualize the differential expression of SDC1 at the mRNA level in TNBC in the GSE62931 dataset.

#### 2.2.2 The assessment of prognostic value

The KM plotter (https://kmplot.com/analysis/) database is a survival analysis tool that allows survival analysis at the mRNA, miRNA, as well as DNA and protein levels, including data from a variety of cancers, such as breast, ovarian, lung and gastric, and liver cancers. The KM plotter database was interrogated to evaluate the association between SDC1 expression levels and clinical prognosis in triple-negative breast cancer patients.

The Human Protein Atlas (HPA) (https://www.proteinatlas.org/) is a database based on proteomics, transcriptomics, and systems biology data that demonstrates gene expression in normal as well as tumor tissues. We employed this database to retrieve the immunohistochemical expression profile of SDC1 in both normal breast tissue and breast cancer.

#### 2.2.3 Function enrichment analysis

The STRING (https://cn.string-db.org/) database is a protein interaction network database based on data from various public databases as well as related literature information, which can be used to retrieve interactions between known proteins and unknown proteins, as well as to provide enrichment analysis of related proteins. Employing the STRING database, we generated a protein-protein interaction (PPI) network centered on SDC1 and identified its top 100 binding partners.

DAVID (https://david.ncifcrf.gov/) is a bioinformatics database that integrates biological data and analytical tools to provide systematic and comprehensive annotated information on biological functions for large-scale genes. he SDC1-associated gene cohort was subjected to functional annotation and pathway enrichment using DAVID's analytical modules.

BioLadder (https://www.bioladder.cn/web/) is an online platform for bioinformatic analysis and visualization of analysis results. We used this online platform to visualize the results of enrichment analysis; we selected the top 10 enrichment results with statistically significant results (p < 0.05) for visualization.

#### 2.2.4 Drug resistance analysis

The GSCA (https://guolab.wchscu.cn/GSCA/) database is a cross-sectional, comprehensive cancer analysis database that includes single-gene, multi-gene, immune infiltration, mutation, and drug sensitivity analyses and contains data on many different types of cancer. Using this database, we evaluated how SDC1 expression levels relate to CTRP drug response.

#### 2.2.5 Molecular docking

PubChem (https://pubchem.ncbi.nlm.nih.gov/) is an open-access database containing data on a large number of chemical substances and biological activities. We used this database to obtain the 3D structure of etoposide.

AlphaFold (https://alphafold.com/) is an open-access database containing a large amount of high-precision protein structure prediction data. We used this database to obtain the protein 3D structure of SDC1.

AutoDockToolS (Version 1.5.7) is a molecular docking software that facilitates the setup, initiation, and analysis of AutoDock runs.In this study, we employed this software to pre-process the small molecule Etoposide and the protein SDC1, and to select appropriate docking sites.

To identify the optimal molecular docking conformations, we employed AutoDock Vina (Version 1.2.3), leveraging its "Iterated Local Search" algorithm, which incorporates continuous local search and repeated iterations.

PyMOL software (Version 3.0.3), a Python language-based 3D structure visualization software, is commonly used for 3D visualization and analysis of biomolecules; Maestro software (Version 13.9.135) is commonly used for the analysis of molecular docking results. We used PyMOL and Maestro to analyze and visualize the docking results of the small molecule Etoposide and protein SDC1.

#### 2.2.6 Immune infiltration analysis

The TIMER 2.0 (http://timer.comp-genomics.org/) database is a database for analysis based on TCGA canceromics data that allows for analytical assessment of tumor immune infiltration using six algorithms. Correlational analysis of SDC1 expression in triple-negative breast cancer and tumor-infiltrating immune cells within the microenvironment was performed using the TIMER 2.0 database.The algorithm that was employed was CIBERSORT, a highly influential deconvolution method that utilizes microarray data with a predefined immune signature matrix to estimate the proportion of 22 tumour-infiltrating immune cells (TIICs) in a given sample. Its core statistical method is Support Vector Regression (SVR), commonly employing Pearson correlation analysis to assess the correlation between expression levels across different immune cell subpopulations.

#### 2.2.7 Single-cell data processing and cluster analysis

We downloaded two datasets (GSE176078, GSE161529) from the GEO database and filtered the data using the R language Seurat package (5.1.0), GSE176078 removing genes that could only be detected in less than 200 cells; GSE161529 also filtered the data using the R language Seurat package, removing genes that could only be detected in less than 200 cells, removing cells with nFeature_RNA (number of genes per cell) ≥ 7000 and ≤ 200, removing cells with nCount_RNA (number of counts per cell) ≥ 30,000 and ≤ 500, and removing percent.mt (percentage of mitochondria-expressed genes) ≥ 10 % of the cells. The data were then normalized and extracted by the “vst” method to find the 2000 genes with the largest variation (highly variable genes). The 2000 highly variable genes were downscaled by PCA to 50 dimensions, and then the effect of downscaling was examined by Elbowplot, and the top 30 dimensions were selected for downstream analysis in this study. Further, unsupervised clustering analysis of cells was done using FindNeighbors and FindClusters functions with the Seurat package, and finally, cells were clustered (resolution = 1) and manually annotated using umap method.A standardized marker gene panel was defined for annotation purposes, with this definition being based on both published literature and the CellMarker 2.0 cell marker database. The classification of a cell cluster as belonging to a specific type is contingent upon the fulfillment of specific criteria. Firstly, the cluster must demonstrate significant overexpression of the classical marker genes of that particular cell type in the FindAllMarkers function analysis (adjusted p-value <0.05, average log2FC > 2). Secondly, it is essential that the cluster does not express or only exhibits weak expression of the marker genes of other major cell types. Box plots of SDC1 expression in various types of cells were plotted using the R language ggplot2 package.

#### 2.2.8 Pseudo-timing analysis

We utilized the monocle (version 2.34.0) package to conduct a mock-timing analysis of three cell types: Cancer cells, Fibroblasts, and Macrophages. We constructed cell mock-timing trajectories using 2000 highly variable genes to sort cells and investigate the dynamics of SDC1 expression.

#### 2.2.9 Cellular communication

Cell-to-cell communication and interactions were analyzed using the CellChat (1.6.1) package, which infers communication probabilities at the signaling pathway level by calculating correlations for all ligand-receptor interactions associated with each pathway and constructing the ligand-receptor network. CellChat uses gene expression data and cell annotations to identify overexpressed genes in each cell class. It models communication probabilities and identifies significant communications by cross-referencing ligand-receptor interaction databases, quantifying cellular communication probabilities using the law of mass action, and inferring statistically and biologically significant cellular communication.

#### 2.2.10 Core aging-related analysis

Following single-cell analysis, examine the expression of target genes in different cell types or subpopulations. Combine the age information of samples with the average expression levels of target genes (calculated within a specific cell subpopulation) to compute Spearman correlation coefficients and significant p-values.

#### 2.2.11 Cell culture

Triple-negative breast cancer cell models (MDA-MB-231 and HCC1086) were procured from the American Type Culture Collection (ATCC). MDA-MB-231 cells were maintained in high-glucose DMEM supplemented with 10 % fetal bovine serum (FBS) and 1 % penicillin-streptomycin antibiotic cocktail. HCC1086 cells were grown in RPMI 1640 medium containing 10 % FBS and 1 % penicillin-streptomycin. All cultures were incubated at 37 °C under 5 % CO_2_ in a humidified atmosphere. Cell lines were routinely verified to be free of mycoplasma, bacterial, and fungal contaminants.

#### 2.2.12 Cell transfection

siRNA-1 was synthesized by Tsingke (Beijing China), while siRNA#2 and siRNA#3 were synthesized by RiboBio (Guangzhou, China). The interference sequences are listed in the following Table 1. Transfections were then performed using the HiPerFect kit (QIAGEN GmbH, Netherlands) in strict accordance with the instructions provided by the kit's manufacturer.

**Table 1:** Sequence of SDC1 siRNA.

siRNA name	Sequence
siRNA-1	5′-AUACUUGUUUCUUGAUCUC-3′
siRNA#2	5′-GGGAGAAUACGGCUGUAGU-3′
siRNA#3	5′-CUUGGAGGAGCCGAAACAA-3′

#### 2.2.13 Real-time fluorescence quantitative PCR

Total RNA isolation was performed with Vazyme's RNA Isolator Total RNA Extraction Reagent (Nanjing, China). Aliquots of purified RNA underwent reverse transcription to cDNA using TranScript Uni All-in-One First-Strand cDNA Synthesis SuperMix for qPCR (TransGen Biotech, Beijing, China). Quantitative real-time PCR (qRT-PCR) amplification was executed following the established protocol, with relative expression levels quantified via the 2^−ΔΔct^ method. Corresponding primer sequences are provided in Table 2.

**Table 2:** Primer sequence for qPCR.

Gene	Primer (Forward)	Primer (Reverse)
SDC1	CGTGGGGCTCATCTTTGCT	TGGCTTGTTTCGGCTCCTC
β-actin	CTCTTCCAGCCTTCCTTCCT	AGCACTGTGTTGGCGTACAG
VEGFA	AACTTTCTGCTGTCTTGG	ACTTCGTGATGATTCTGC

#### 2.2.14 Angiogenesis experiment

After HUVECs were cultured under different conditions of medium for 24 h, they were inoculated into 96-well plates, and endothelial cell tubulation was photographed and analyzed using Image J for endothelial cell tubulation according to 2 × 10^4^ cells per well at 37 °C, 5 % CO2 for 8 h.

#### 2.2.15 TEM

For TEM imaging, Samples were applied to glow-discharged formvar/carbon-coated grids (EMS) and imaged via JEM-F200 TEM configured at 80 kV.

#### 2.2.16 EDS

For EDS imaging, specimens were applied to glow-discharged Formvar®/carbon-coated grids (Electron Microscopy Sciences) and visualized using an Ultim Max 80 system.

#### 2.2.17 Laser confocal imaging

The precise number of cells was inoculated onto a specialized confocal culture dish (BS-20-GJM). The precise concentration of AuCl4-precursor was introduced into the culture dish in conjunction with SDC1 shRNA. Following the formation of gold nanocluster-RNA complexes *in situ*, fluorescence micrographs were acquired via laser confocal microscopy with 488 nm excitation. Photographed using a laser confocal microscope (Lcica/STELLARIS 5, Germanny)

#### 2.2.18 Raman spectroscopy

For SERS spectroscopy studies, samples were deposited on a flat segment of aluminum foil and analyzed with a laser microscopic Raman spectrometer (Thermo Fisher Scientific, dxr3).

Fourier transform infrared spectrum, FT-IR.

For FTIR spectroscopy studies, samples were dried at a low temperature and then pressed with potassium chloride. With the use of infrared spectrometry (Thermo Fisher Scientific,is50), measurements were taken immediately in 100 w of power.

#### 2.2.19 Statistical analysis

Statistical analyses were conducted with GraphPad Prism 10. Survival curves were compared via log-rank testing, while Spearman's rank-order correlation assessed relationships between SDC1 expression and immune infiltration levels. Inter-group comparisons utilized Student's two-tailed *t*-test, with statistical significance defined as p < 0.05.

## 3 Results

**Fig. 1:**
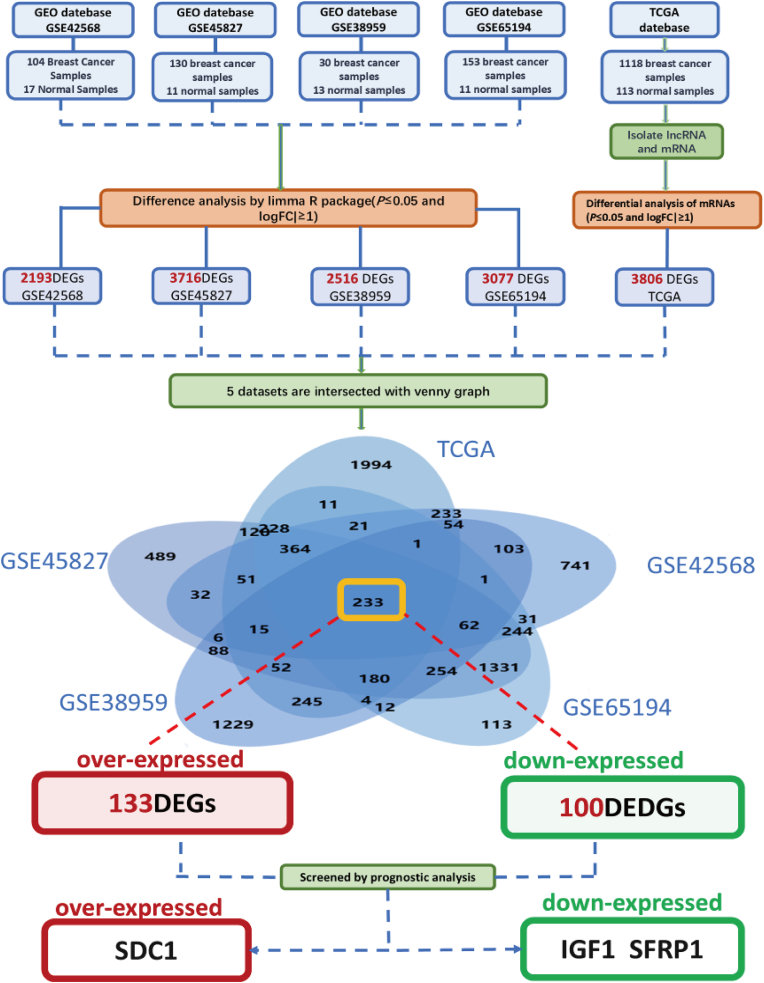
Process of genetic screening with data from TCGA and GEO databases (GSE42568, GSE65194, GSE38959, GSE45827).

Following the download of the datasets GSE42568, GSE65194, GSE45827, and GSE38959 from the GEO database, a total of 417 breast cancer and 52 normal breast were obtained. Subsequent analysis focused on the screening of genes that exhibited differential expression in breast cancer tissues, with the specific screening process outlined below (Fig. 1): Differential gene expression analysis via limma (R package) revealed transcriptomic alterations distinguishing malignant breast specimens from normal controls, applying thresholds of |log_2_FC| ≥ 1 and adjusted *P* ≤ 0.05.There are 2193 DEGs in GSE42568; 3077 DEGs in dataset GSE65194; 3716 DEGs in dataset GSE45827; and 2516 DEGs in dataset GSE38959. In combination, breast cancer expression data mined from the TCGA database (including 1118 breast cancer and 113 normal breast) were used to isolate lncRNAs and mRNAs; the mRNAs were analyzed using the limma R package for DEGs (|logFC| ≥ 1 and *P* ≤ 0.05) in breast cancer versus normal tissues, and a total of 3806 DEGs were screened. The intersection of the aforementioned five datasets yielded 233 genes, 133 of which were found to be up-regulated in tumors, and 100 of which were found to be down-regulated. The correlation of these genes with the prognosis of TNBC was then analyzed separately. This analysis identified three genes that exhibited a correlation with the prognosis of triple-negative breast cancer. these genes, Insulin Like Growth Factor 1 (IGF1) and Secreted Frizzled Related Protein 1 (SFRP1), were found to be down-regulated in tumor tissues, while the third gene, SDC1, also known as Syndecan 1, was found to be up-regulated in tumor tissues.

IGF-1 (insulin-like growth factor-1), a peptide signaling molecule, coordinates essential functions including organismal development, mitogenesis, and metabolic stability maintenance [22]. The encoded protein is structurally similar to insulin, and it activates downstream signaling pathways by binding to receptors such as IGF1R [23]. Current research on triple-negative breast cancer includes anti-tumor-related pathways against IGF1 [24], promotion of tumor cell death [25], and targeting the IGF-1/IGF-1R signaling axis for the treatment of TNBC [26].

SFRP1 belongs to the SFRP protein family, which exhibits a dual function: it inhibits the Wnt pathway (acting as a tumor suppressor) and promotes tumor progression under specific conditions [27]. Current research on TNBC indicates that SFRP1 expression is frequently silenced due to promoter hypermethylation in this condition. This aberrant activation of the Wnt/β-catenin pathway subsequently leads to uncontrolled tumor metastasis [28]. Notably, SFRP1 deletion does not require the involvement of the Wnt pathway, resulting in chemotherapy resistance [29].

Despite documented SDC1 overexpression in TNBC and its established role in triggering c-Src/FAK signaling to drive cell migration, its specific pathogenesis, chemoresistance, single-cell analysis, and design of delivery systems have not been reported [[19], [20], [21]]. Consequently, this study has focused on SDC1 as the primary object of investigation.

### 3.1 High SDC1 expression characterizes triple-negative breast cancer and is correlated with compromised patient prognosis

**Fig. 2:**
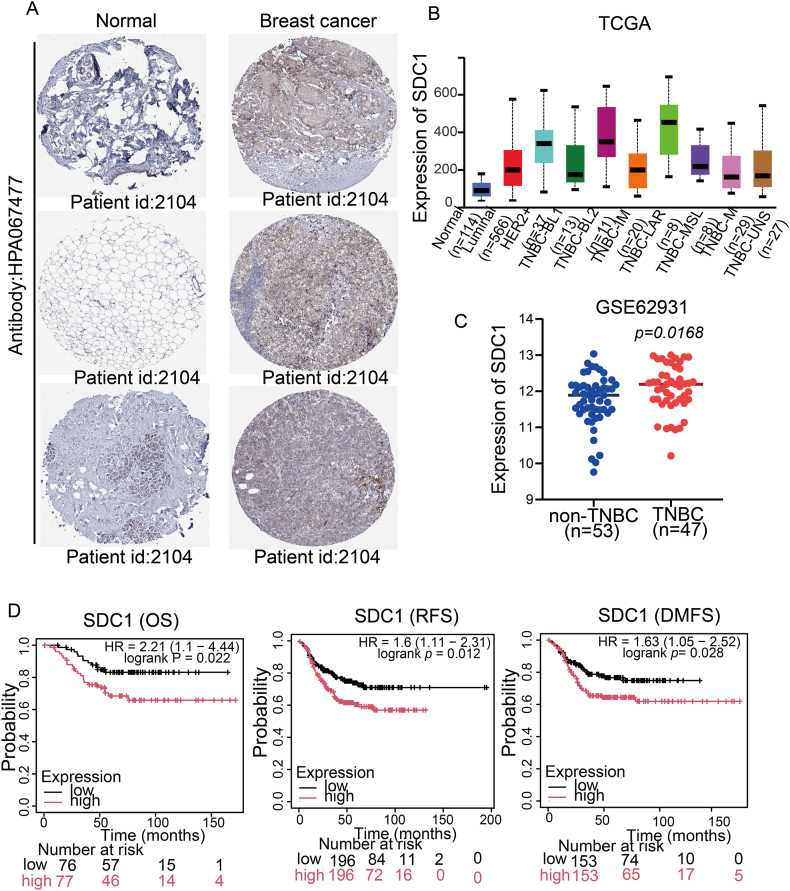
SDC1 is expressed at high levels in TNBC and is significantly associated with poor patient prognosis.A. Protein level expression of SDC1 in breast cancer tissues (immunohistochemistry images obtained from the HPA online database); B. Expression levels of SDC1 in different molecular subtypes of breast cancer with the help of the TCGA dataset; C. Analysis of SDC1 expression levels in TNBC and non-TNBC (Her2+ type, Luminal A type,Luminal B type) in the GEO database. D. The prognostic impact of SDC1 on TNBC patients will be evaluated, including OS, RFS and DMFS, with the cutoff value set at the median.

To gain enhanced insight into the SDC1 expression landscape in breast cancer, multi-omics data were subjected to systematic interrogation. Immunohistochemical data from the Human Protein Atlas (HPA) revealed markedly elevated SDC1 protein expression in breast carcinoma specimens compared to normal mammary tissues (Fig. 2A). In the downstream analytical phase of the transcriptome data from the UALCAN platform for TCGA breast cancer revealed that the expression of SDC1 mRNA was significantly up-regulated in TNBC compared with other molecular subtypes (Luminal A/B, HER2+ sex) (Fig. 2B).

We sought to re-examine SDC1 expression in distinct breast cancer subtypes in this study In pursuit of this objective, the GSE62931 dataset (n = 100) was integrated in GEO, and SDC1 expression was significantly up-regulated in TNBC samples (n = 54) compared to non-TNBC specimens (n = 47, inclusive of Luminal and HER2+ subtypes), as evidenced by the results (Fig. 2C). The findings from the present study suggest that SDC1 might be able to serve as a diagnostic indicator for TNBC.

In order to evaluate the prognostic value of SDC1 in TNBC, a survival analysis was performed based on the Kaplan-Meier Plotter database. Significantly inferior OS, RFS, and DMFS were observed in the SDC1-high group through prognosis analysis (Fig. 2D).Preliminary data indicate that high SDC1 expression is associated with a trend toward poorer survival (P < 0.05), but its independent prognostic value requires confirmation in future studies incorporating comprehensive clinical information. Subsequent analyses will focus on elucidating the underlying mechanisms through which SDC1 contributes to adverse outcomes in breast cancer patients.

### 3.2 Analysis of related downstream pathways involved in SDC1

**Fig. 3:**
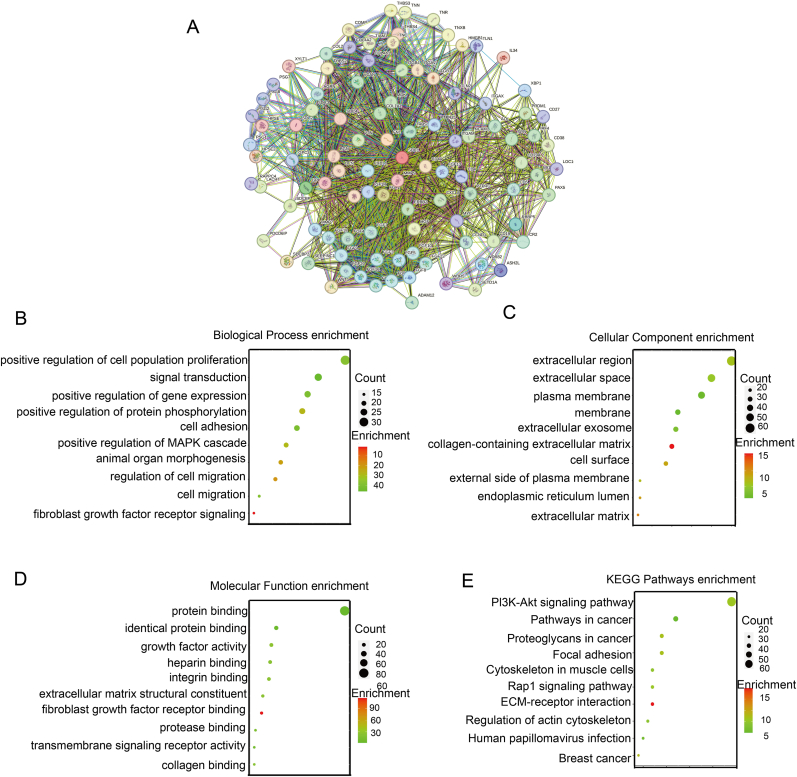
GO enrichment analysis and KEGG enrichment analysis of SDC1 interacting proteins. A. Interacting PPI of SDC1; B.-D. The following analyses were conducted: GO enrichment analysis, enrichment analysis of Biological Processes, Cell Components, and Molecular Functions. The size of the circle indicates the number of enriched genes, and the color indicates the enrichment degree. The number of enriched genes is indicated by the size of the circle, and the shade of color indicates the degree of enrichment. E. The KGEE enrichment analysis. The size of the bubble indicates the number of enriched genes, and the shade of color indicates the degree of enrichment. (For interpretation of the references to color in this figure legend, the reader is referred to the Web version of this article.)

In order to further explore the relevant functions and specific mechanisms of SDC1, the STRING database was utilized to identify genes that interact with SDC1. From these data, the top 100 genes were selected to construct a protein network (PPI) of interactions with SDC1 (Fig. 3A). Consequently, we proceeded to employ the DAVID online database to subject the aforementioned 100 genes to both GO and KEGG pathway analysis. The findings of biological process (BP) analysis suggested a potential involvement of SDC1 in tumor proliferation, metastasis, fibroblast growth factor receptor signaling pathway, and positive regulation of protein phosphorylation (Fig. 3B); Molecular function (MF) enrichment analysis indicated that proteins interacting with SDC1 might bind to fibroblast growth factor receptor, transmembrane proteins, and extracellular matrix structures (Fig. 3D). Furthermore, cellular component (CC) analysis demonstrated that proteins interacting with SDC1 primarily affected cell membranes, the extracellular matrix, and exosome-related structures. (Fig. 3C); Analysis via KEGG pathway enrichment demonstrated that Rap-1 signaling pathway, ECM-receptor interactions, the PI3K-Akt signaling pathway, as well as breast cancer-associated pathway were the primary sites of enrichment for the relevant proteins.(Fig. 3E). Consequently, based on the aforementioned results, SDC1 may be closely related to the fibroblast growth factor (FGF) family and its associated pathways.

### 3.3 SDC1 enhances therapeutic resistance to etoposide in TNBC patients

Patients diagnosed with TNBC typically have a significantly poor prognosis, and their limited therapeutic response is primarily attributed to the susceptibility of tumor cells to drug resistance [9]. Research has demonstrated that SDC1 fosters cisplatin resistance in hepatocellular carcinoma cells via the PI3K-AKT pathway [30]. Its impact on tumor chemoresistance has also been documented in colorectal cancer [31] and prostate cancer [32]. However, there is a paucity of research addressing the potential impact of SDC1 on chemoresistance in TNBC.

**Fig. 4:**
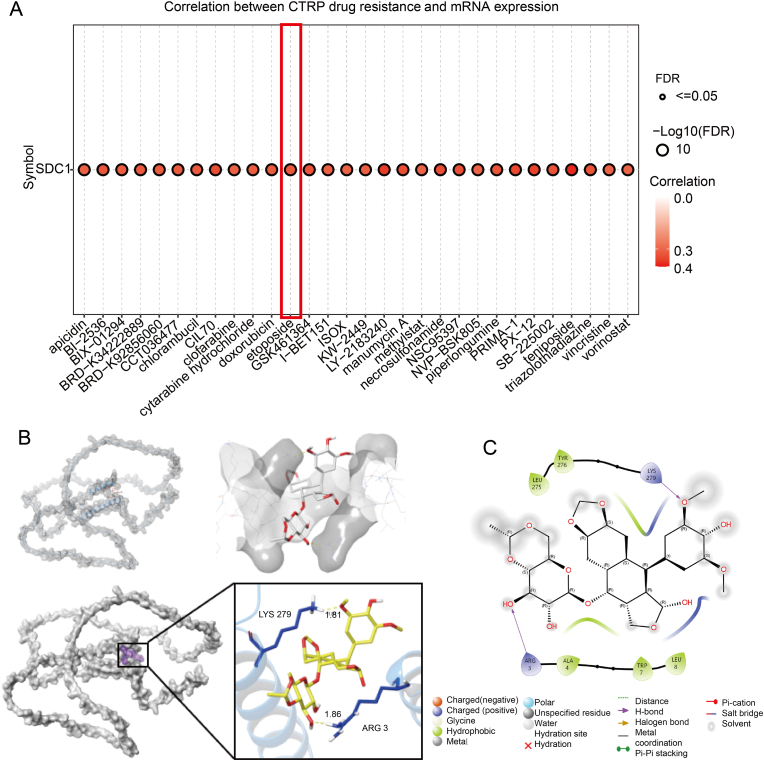
Drug resistance analysis of SDC1. A. SDC1 and CTRP drug resistance analysis, red color represents positive correlation. B. Molecular docking of SDC1 with etoposide (binding energy −7.6 kcal/mol), diagram of small molecule-protein surface interactions and names of amino acid residues for hydrogen bonding interactions. The blue part is the protein and the yellow part is the small molecule. C. 2D diagram of the interaction of etoposide with the SDC1 protein. (For interpretation of the references to color in this figure legend, the reader is referred to the Web version of this article.)

In order to explore this question, we conducted an analysis of SDC1 resistance to FDA-approved drugs using the GSCA online database and reviewed the Triple Negative Breast Cancer Treatment Guidelines from 2024 [33]. Elevated SDC1 expression correlated positively with acquired etoposide resistance in our analysis (Fig. 4A). As a classical chemotherapeutic agent, etoposide inhibits DNA topoisomerase II to exert antitumor activity. It serves as a key therapeutic for diverse malignancies [34,35] and is currently employed as monotherapy in advanced triple-negative breast cancer patients [36]. Furthermore, our molecular docking using Autodock4 showed that the binding energy between Etoposide and SDC1 was −7.6 kcal/mol, and the interaction forces, including hydrogen bonding and hydrophobic forces helped to maintain the stability of their complex, where the amino acids forming hydrogen bonding interactions with Etoposide are Arg 3, Lys 279. The amino acids forming hydrophobic interactions with etoposide are Ala 4, Trp 7, Leu 8, Leu 275, and Tyr 276 forming hydrophobic interactions (Fig. 4B and C). Molecular docking is a computer simulation-based technique for predicting biomolecular interactions. The primary objective involves deciphering biophysical interaction mechanisms through computational determination of ligand-receptor binding conformations and energetics [37,38]. The utilization of computational modelling techniques has facilitated the prediction of a potential binding interaction between SDC1 and etoposide. In order to validate the interaction between etoposide and SDC1, cellular thermodynamic shift analysis (CETSA) was employed in order to assess the effect of etoposide on SDC1 thermal stability within cells. The experimental findings demonstrated that, in comparison with the control group, etoposide treatment significantly enhanced SDC1 thermal stability (Figure S2 A-B). This observation suggests that the binding between the two occurs in the cellular environment.

In order to further test this hypothesis at the functional level, SDC1 was knocked down and overexpressed in cells, after which alterations in the IC_50_ values for etoposide were evaluated. The results showed that compared to the control, SDC1 knockdown led to a significant about 50 % decrease in the etoposide IC50, whereas SDC1 overexpression resulted in a about 80 % increase (Figure S2 C). These findings suggest that targeting SDC1 may enhance chemotherapy sensitivity in patients diagnosed with triple-negative breast cancer, thereby improving their prognosis.

### 3.4 Analysis of immune cell infiltration

SDC1 may promote tumorigenesis by promoting cellular senescence and, in turn, tumorigenesis.

**Fig. 5:**
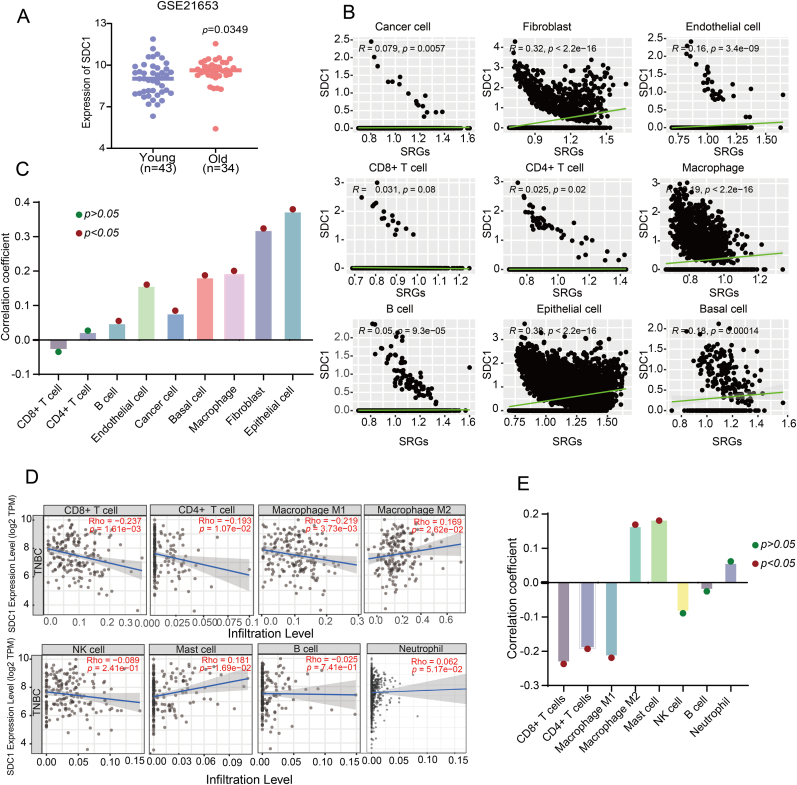
Correlation of SDC1 with senescence of different cells in the tumor microenvironment and analysis of immune cell infiltration in triple-negative breast cancer. A. Expression levels of SDC1 in triple-negative breast cancer patients of different ages (defined as young <60 years old (n = 43)and >60 years old(n = 34)) B. Correlation analysis of SDC1 with senescence of different cells in the tumor microenvironment (Epithelial cell, Cancer cell, Fibroblast, B cell, Macrophage, Endothelial cell, CD4^+^ T cell, CD8^+^ T cell, Basal cell).C. Statistical graph of correlation analysis between SDC1 and senescence of different cells in tumor microenvironment. D. Correlation analysis between SDC1 and correlation analysis of immune cell infiltration (CD8^+^ T cells (Cor = -0.237, *p* < 0.05), CD4^+^ T cells (Cor = -0.193, *p* < 0.05, Macrophage M1 (Cor = -0.219, *p* < 0.05), Macrophage M2 (Cor = 0.169, *p* < 0.05), Mast cell (Cor = 0.181, *p* < 0.05) Neutrophil (Cor = 0.062, *p* > 0.05, NK cell (Cor = −0.089, *p* > 0.05) B cell (Cor = −0.025, *p* > 0.05)).E. Correlation analysis between SDC1 and immune cell infiltration Statistical graph.

While mining the dataset of GEO data, we found that the expression level of SDC1 was relatively high in older patients (age >60 years) compared to younger (age <60 years) TNBC patients in the dataset GSE21653 (Fig. 5A). We further analyzed the correlation between SDC1 and cellular senescence in the tumor microenvironment of TNBC. The results showed (Fig. 5B and C) that SDC1 was significantly correlated with fibroblast and mammary epithelial cell senescence (cancer cell Cor = 0.079 *p* = 0.006; fibroblast Cor = 0.321 *p* < 0.0001; endothelial cell Cor = 0.159 *p* < 0.0001; CD8^+^ T cell Cor = −0.031 *p* = 0.08; CD4^+^ T cell Cor = 0.025 *p* = 0.020; macrophage Cor = 0.190 *p* < 0.0001; B cell Cor = 0.050 *p* < 0.0001; epithelial cell Cor = 0.375 *p* < 0.0001; basal cell Cor = 0.183 *p* < 0.0001). This suggests that SDC1 may not only be used as a risk factor for triple-negative breast cancer in the elderly, but also by inducing cellular senescence in the TME.

Therefore, we investigated the potential impact of SDC1 on the immune microenvironment of triple-negative breast cancer. To this end, the TIMER 2.0 online database was utilized to analyze the relationship between SDC1 and immune cell infiltration in triple-negative breast cancer (Fig. 5D and E). The results demonstrated a negative correlation between SDC1 expression and T-cell infiltration of CD8^+^ T cells (Cor = −0.237, *p* < 0.05) and CD4^+^ T cells (Cor = −0.193, *p* < 0.05) The present study found a negative correlation between SDC1 and the infiltration of macrophages of the M1 type, as well as a positive correlation between SDC1 and the infiltration of macrophages of the M2 type. The correlation coefficients for these relationships were −0.219 and 0.169, respectively, and both were found to be statistically significant (*p* < 0.05). SDC1 was positively correlated with mast cell and neutrophil infiltration. Mast cell (Cor = 0.181, *p* < 0.05) Neutrophil (Cor = 0.062, p > 0.05); SDC1 expression was negatively correlated with infiltration of NK cell (Cor = -0.089, *p* > 0.05) B cell (Cor = -0.025, *p* > 0.05).

### 3.5 Decoding SDC1 dynamics in distinct tumor-infiltrating cell types through single-cell interrogation

These findings demonstrated SDC1's involvement in immune cell recruitment within the tumor niche. Subsequently, we implemented high-resolution single-cell profiling of SDC1 expression across malignant and stromal compartments. Single-cell analysis is a research method that utilizes high-throughput technology to accurately analyze the gene expression, protein composition, epigenetic features, or metabolic status of individual cells. This approach enables the revelation of heterogeneity among cells and the expression of genes in different cells, providing a novel perspective for the study of tumor mechanisms and precision therapy [[39], [40], [41]].

For mapping SDC1 expression heterogeneity within TNBC cellular compartments, the single-cell data of TNBC from the GEO database was mined, resulting in the acquisition of two datasets, GSE176078 (9 TNBC samples) and GSE161529 (4 TNBC samples). Subsequent to filtration and analysis by clustering, the cell clusters were obtained by manual annotation.

**Fig. 6:**
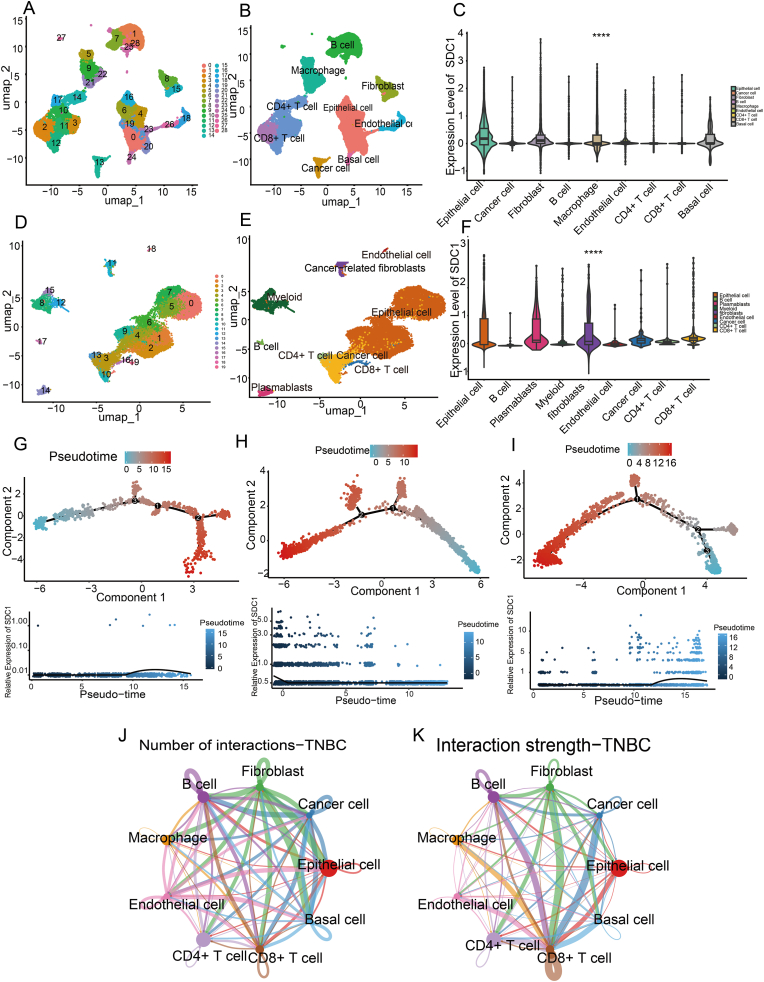
Single-cell data analysis of SDC1. A-C. Analysis of dataset GSE176078 (A, clustering of cells using the umap method (resolution = 1)); B, annotated UMAP clustering plots; C.Expression levels of SDC1 in various cell types (Epithelial cell, Cancer cell, Fibroblast, B cell, Macrophage, Endothelial cell, CD4^+^ T cell, CD8^+^ T cell, Basal cell); D-F. Dataset GSE161529 analysis (D, clustering of cells using the umap method (resolution = 1)); E., Annotated UMAP clustering plot; F. SDC1 expression levels in various cell types (Epithelial cell, Cancer cell, Cancer-related fibroblast, B cell, Myeloid, Endothelial cell, CD4^+^ T cell, CD8^+^ T cell, Plasma blasts)); G-I pseudo-temporal analysis (G, Cancer cell dynamics in response to SDC1 expression levels, H., Macrophage dynamics in response to SDC1 expression levels, H, Fibroblast dynamics in response to SDC1 expression levels); J-K cellular communication (J, number of cell-cell interactions, K, strength of cell-cell interactions).The Y-axis displays log-normalized expression levels.

In the dataset GSE176078 we obtained 29 cell clusters by filtering the data, dimensionality reduction analysis, and then clustering the cells using the Uniform Manifold Approximation and Projection (UMAP) method (Fig. 6A), and further manually annotating the resulting cell clusters, and finally obtaining 9 clusters of cells respectively were Epithelial cell, Cancer cell, Fibroblast, B cell, Macrophage, Endothelial cell, CD4^+^ T cell, CD8^+^ T cell, Basal cell (Fig. 6B). In the dataset GSE161529 we obtained 20 cell clusters by filtering the data, downscaling the analysis, and then clustering the cells using the UMAP method (Fig. 6D), and further manually annotated the resulting cell clusters, which finally resulted in 9 cell clusters of Epithelial cell, Cancer cell, fibroblast, B cell, Myeloid, Endothelial cell, CD4^+^ T cell, CD8^+^ T cell, Plasma blasts (Fig. 6E). Subsequently, we analyzed the expression of SDC1 in various cells and used the R language ggplot2 package to draw box line plots of SDC1 expression in various types of cells (Fig. 6C–F). The results showed that SDC1 was expressed at high levels in fibroblasts, that indicates statistically significant relative enrichment of its expression level among the nine identified cell types.

### 3.6 Pseudo-temporal analysis of SDC1 succession in different cells

Given the established role of SDC1 in promoting cellular senescence, this study examined the temporal progression of SDC1 gene expression using pseudo-temporal sequencing. This computational approach infers developmental hierarchies by tracking gene expression evolution in heterogeneous cell subsets across pseudo-time [42]. Utilizing this approach, we sought to elucidate the potential evolutionary sequence between cell states of fibroblasts, macrophages, and cancer cells by leveraging the continuity feature of SDC1 gene expression. To this end, cellular pseudo-temporal trajectories were constructed by sequencing cells using 2000 highly variable genes. The results demonstrated the dynamics of cellular and gene SDC1 over time (Fig. 6G–I). The results showed that with changes in SDC1 expression, fibroblasts, macrophages, and cancer cells all showed different differentiation nodes. Consequently, we hypothesize that SDC1 may function as a pivotal gene influencing the proliferation and progression of fibroblasts, macrophages, and cancer cells within the tumor microenvironment of TNBC. We further propose that SDC1 may regulate cellular functions within the tumor by modulating the differentiation of fibroblasts and macrophages.

### 3.7 SDC1 is involved in extensive cellular communication in the tumor microenvironment

In light of these findings, we subsequently conducted an exhaustive examination of the potential communication pathways between cells within the tumor microenvironment of TNBC (Fig. 6J and K). The analysis of cell communication can facilitate our understanding of cell-cell interactions and the resolution of intercellular communication networks. This analysis can reveal the interactions of various types of cells during development, explore the tumor immune microenvironment, and uncover potential therapeutic targets for the disease [43,44]. The findings indicate the presence of extensive interactions among distinct cell types within the tumor microenvironment of TNBC, including epithelial cells, cancer cells, B cells, myeloid cells, endothelial cells, CD4^+^ T cells, CD8^+^ T cells, and plasma blasts.

### 3.8 Construction of GNCs-SDC1 shRNA *in situ* nucleic acid delivery system based on bioinformatics analysis

It has been demonstrated that SDC1 is associated with immune infiltration and angiogenesis, as well as etoposide resistance. Consequently, if it is feasible to utilize nucleic acid delivery to achieve a reduction in the expression level of SDC1 in tumor cells. This discovery paves the way for developing targeted clinical interventions against TNBC [45].

Therapeutic nucleic acids have two main modes of action: up-regulation of target genes (DNA plasmid vectors containing mRNA expression sequences, mRNAs, or miRNAs) [46] and down-regulation of target genes (DNA plasmid vectors containing shRNA expression sequences, siRNAs) [47] These molecules hold considerable promise in the realm of tumor-related molecular mechanism research, with numerous in vivo and in vitro experiments involving the expression and knockdown of DNA or RNA [48,49]. Gene therapy demonstrates considerable promise in the treatment of tumors [50]. The advent of the first mRNA vaccine for the prevention of the novel strain of severe acute respiratory syndrome (SARS-CoV-2) in 2021 further underscores the societal value of gene therapy [51].

In this study, the *in situ* self-reactive GNCs [46] innovatively proposed by our group were utilized to achieve SDC1 shRNA targeted delivery. Specifically, the reducing microenvironment at the tumor site was exploited to reduce the GNCs precursor to a GNCs, which can form a complex with SDC1 shRNA at the tumor site to achieve targeted delivery of SDC1 shRNA at the tumor site.

Initially, three siRNAs targeting SDC1 were procured, and their transfection efficiency was evaluated using commercially available transfection reagents, Neofect. As depicted in Fig. S1, the third sequence exhibited higher transfection efficiency, thus being selected for further development. Notably, shRNA have been shown to exhibit enhanced stability in comparison to siRNA [47]. Consequently, the circulation time of shRNA in the body is prolonged.

**Fig. 7:**
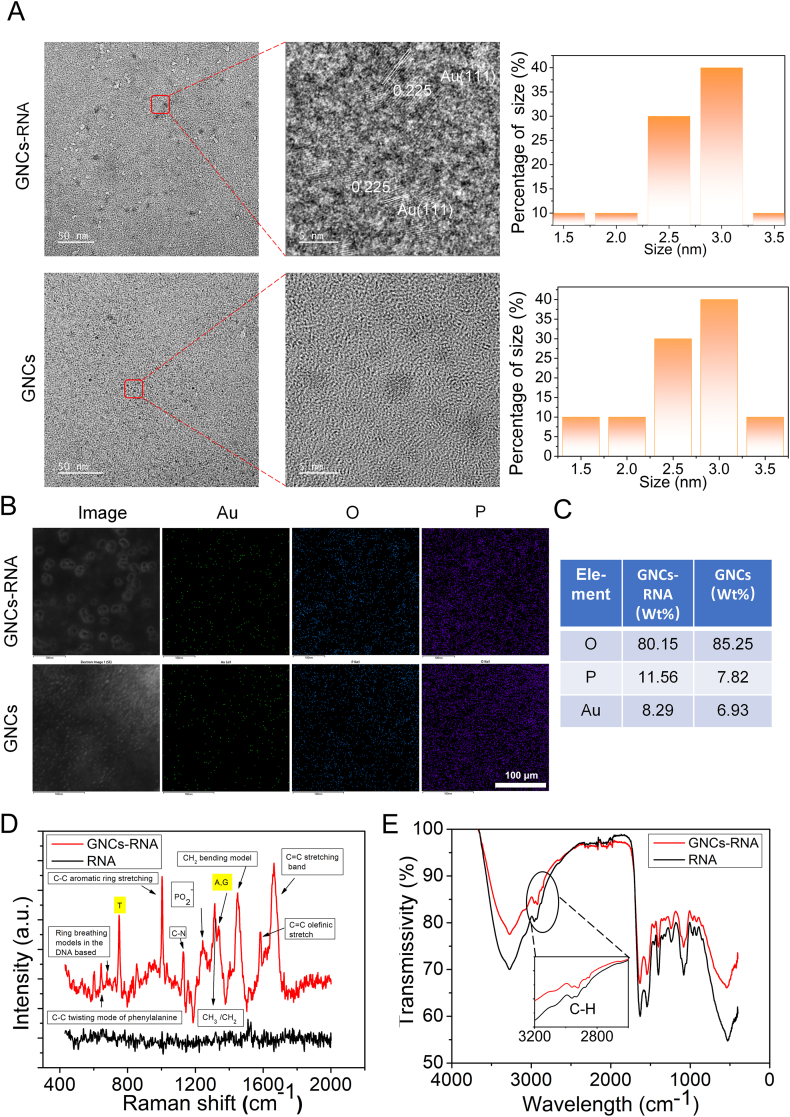
Characterization of GNCs-RNA complexes. A. TEM image in *in situ* self-reactive GNCs; the size of gold nanoclusters in GNCs-RNA complexes are shown on the right. Figure B shows the EDS-mapping of the three elements Au, O, P. Figure C shows the mass ratio of GNCs in different groups. The Raman and FTIR spectra of GNCs-RNA complexes and controls are shown in Figures D and E, respectively. (For interpretation of the references to color in this figure legend, the reader is referred to the Web version of this article.)

Following the fracture of the cells through physical freezing and thawing, the cytoplasmic precipitation was subjected to TEM (Fig. 7A). It was observed that gold nanoclusters were formed in the GNCs group, as well as in the GNCs and SDC1 shRNA groupings. The size of all of these clusters was found to be approximately 3 nm. To facilitate the subsequent discussion, the group containing GNCs and SDC1 shRNA was designated as GNCs-RNA complex. The TEM results indicate that the GNCs in this group exhibit a higher degree of agglomeration compared to the group with GNCs added alone. This observation suggests that the GNCs were specifically brought together after the physical interaction of the GNCs with the shRNA. The high-resolution TEM results can be more intuitively seen in the formation of the GNCs-shRNA complexes. Furthermore, the lattice spacing of the nanoclusters was found to be 0.225, which is consistent with the Au (111) crystal plane [52].

The EDS mapping (Fig. 7B) and EDS atomic mass ratio (Fig. 7C) results demonstrate that the GNCs-RNA sample contains a greater number of gold elements compared to the GNCs group. This phenomenon likely stems from RNA-templated synthesis, enhancing reduction efficiency of gold nanoclusters (GNCs). Conversely, the EDS mapping results indicate that the GNCs-RNA complex group contains more P elements, suggesting that the GNCs-RNA complex possesses a greater abundance of shRNA enriched on GNCs compared to the GNCs group. Furthermore, Fig. 7C shows that compared to the GNCs group, the enrichment rate of gold nanoclusters in the GNCs RNA complex group increased by 19.62 % in terms of the mass ratio of gold elements, indicating that the reducing ability of nucleic acids is a key determining factor in this process.

The results of the Surface enhanced Raman scattering (SERS) detection (Fig. 7D) demonstrated that the Raman signal of the GNCs-RNA complex group was enhanced by more than 1000-fold in comparison with that of the control group. This finding indicates that the *in situ* self-reactive gold nanoclusters present within the complex exhibit a strong Raman enhancement effect. Furthermore, the unique structures present in nucleic acids, such as thymine (746 cm^−1^), adenine, and guanine (1337 cm^−1^), were observed in the GNCs-RNA complex group. Additionally, ring breathing modes in DNA bases (679 cm^−1^) were detected. In addition, the C-C twisting mode of phenylalanine (645 cm^−1^), C-C aromatic ring stretching (1002 cm^−1^), C=C olefinic stretch (1585 cm^−1^), C=C stretching band (1667 cm^−1^), and other carbon-carbon bond twists were also observed. Furthermore, the C-N (1127 cm^−1^) and C-H bonds (1309 cm^−1^, 1447 cm^−1^) are more clearly defined, and the position of PO_2_^−^ (1243 cm^−1^) is more significant. The C-H bond fluctuations observed in the Fourier transform infrared spectroscopy (FTIR) spectra (Fig. 7E), which can be assisted with the Fourier Raman spectra, proved that the physical interaction of GNCs with nucleic acids appeared in the GNCs-RNA complexes, which further proved the formation of the GNCs-RNA complexes. One possible reason for Raman enhancement is the chemical enhancement mechanism, which involves charge transfer interactions between gold nanoclusters and adsorbed molecules, altering molecular vibration modes and increasing Raman scattering cross sections.

### 3.9 Successful intracellular delivery of GNCs-SDC1 shRNA by *in situ* nucleic acid delivery system

**Fig. 8:**
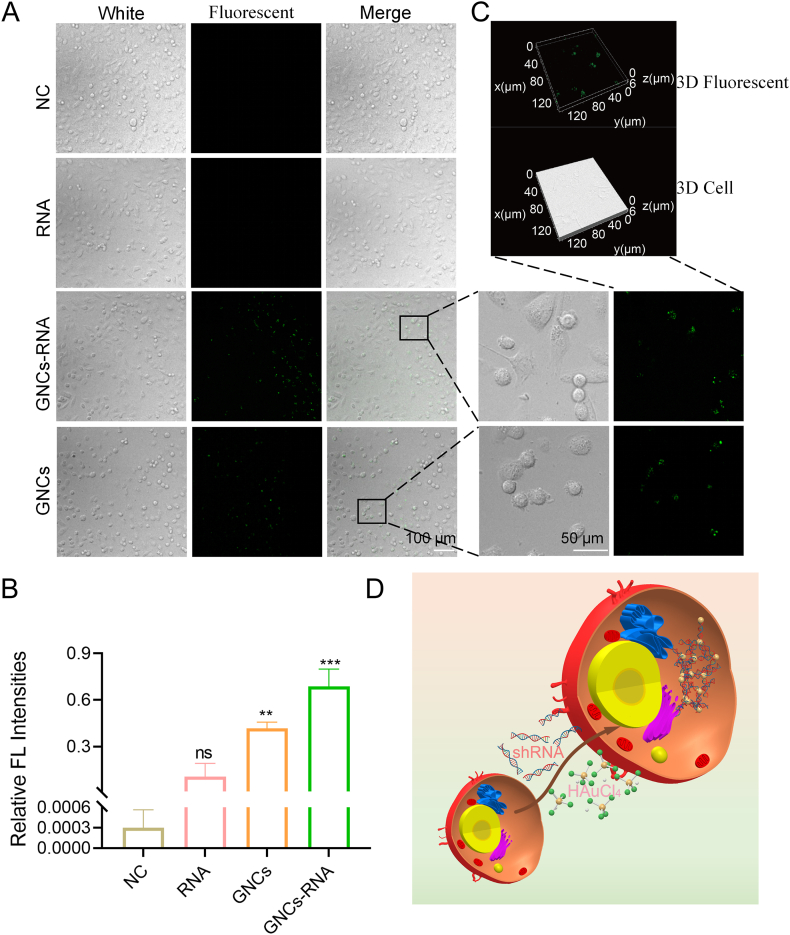
Demonstration of fluorescence imaging of tumor cells by GNCs-RNA complexes with the aid of laser confocal microscopy. A. Image. B. Fluorescence intensity statistics results.C.3D Image D. Scheme for GNCs-RNA complex synthesis. Magnification 200×, scale 100 μm, magnification 800×, scale 500 μm ∗*p* < 0.05, ∗∗*p* < 0.01, ∗∗∗*p* < 0.001.

Given the strong fluorescence targeting of *in situ* self-reactive gold nanoclusters observed in previous results using laser confocal fluorescence microscopy, we examined the targeting of GNCs-RNA complexes with TNBC cells (Fig. 8A–B). The formation of additional gold nanoclusters appeared to enhance this targeting, as evidenced by the stronger fluorescence observed in the GNCs-RNA complexes under 450 nm excitation light compared to the GNCs group (Fig. 8A). As illustrated in Figure C, the confocal fluorescence 3D image, the majority of the fluorescence is observed within the cell rather than surrounding the cell membrane. This observation further supports the hypothesis that the GNCs may have entered the cell through cytosis. In summary, we have demonstrated that, within the highly reducing microenvironment at the tumor site, the GNCs precursor undergoes reduction to form GNCs. Furthermore, we have shown that shRNA can be utilized as a template to enhance this process at the tumor site. Additionally, the co-delivery of GNCs and shRNA results in the formation of GNCs-RNA complexes (Fig. 8D). This method is both green and convenient, with significant potential for clinical application.

### 3.10 The *in situ* nucleic acid delivery system has been shown to exert an anti-angiogenic effect

Firstly, in order to validate the specificity and reliability of SDC1 knockdown, non-overlapping shSDC1 and siSDC1#2 sequences were employed for knockdown. The efficacy of the knockdown was confirmed at both the mRNA and protein levels via qRT-PCR and Western blot analysis (Fig. S3). In the subsequent stage of the research, the potential mechanisms of SDC1 in angiogenesis were explored. To this end, changes in the transcription and translation levels of the key angiogenic mediator VEGFA [53] under SDC1 silencing and overexpression conditions were examined. The results demonstrated that the knockdown of SDC1 led to a significant decrease in VEGFA expression at both the mRNA and protein levels. Conversely, the expression of SDC1 was found to be markedly increased in response to SDC1 overexpression (Fig. S3).

In order to establish genetic evidence for SDC1 function, SDC1 re-expression experiments were performed. The reintroduction of SDC1 expression in GNCs-RNA cells led to a significant reversal of the inhibition of angiogenesis caused by SDC1 knockdown (Fig. S4), providing robust experimental support for SDC1's role in angiogenesis. However, further research is required to confirm its vasculogenesis-promoting effects within the body.

**Fig. 9:**
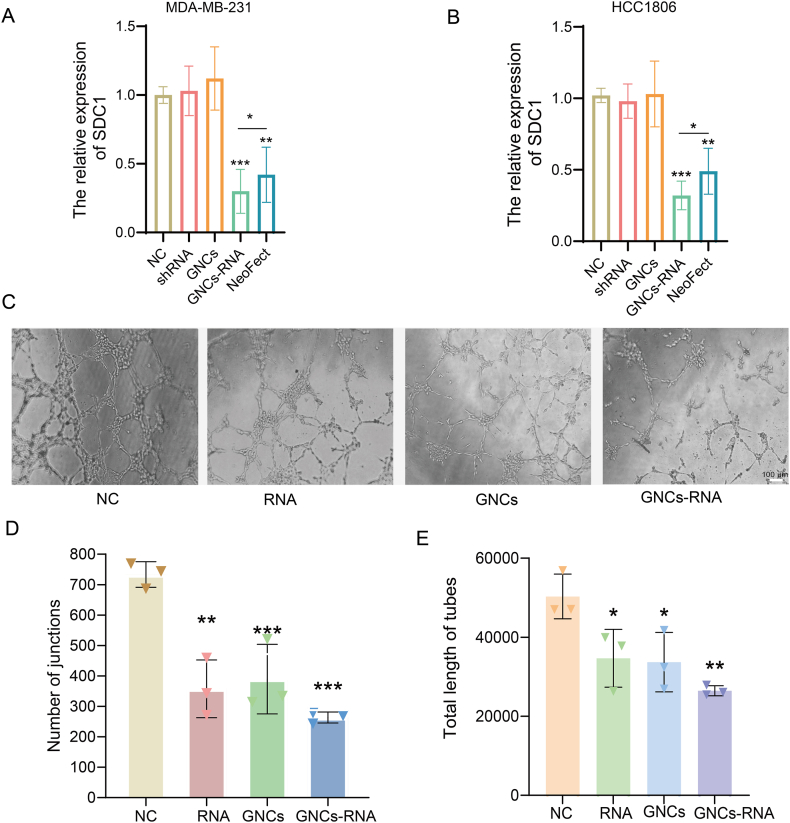
GNCs-RNA complexes have significant nucleic acid delivery capacity. *In situ* self-reactive GNCs-RNA complexes have greater nucleic acid delivery capacity compared with commercial transfection reagents in A and C. Delivering SDC1 shRNA inhibits angiogenesis in TNBC cell line (MDA-MB-231). D-E. Analysis of angiogenic capacity. The number and total length of tube connections indicate the angiogenic capacity of each group. Magnification 100×, scale bar 100 μm ∗*p* < 0.05, ∗∗*p* < 0.01, ∗∗∗*p* < 0.001, ∗∗∗∗*p* < 0.0001.

This work pursued mechanistic evidence that SDC1 silencing with shRNA vectors disrupts tumor vascularization. The initial step in this investigation involved the incorporation of SDC1 shRNA and gold nanocluster precursors into the MDA-MB-231. Subsequently, the conditioned medium from the breast cancer cells was collected and utilized to culture human umbilical vein endothelial cells (HUVEC) for the tube formation assay. The demonstrated that the delivery of SDC1 shRNA effectively suppressed the HUVEC (Fig. 9C–E).

## 4 Discussions

TNBC is a highly aggressive form of the disease with the worst prognosis. The dearth of effective therapeutic options and the poor prognosis associated with TNBC present a significant challenge in the effective management of this highly lethal form of cancer. The development of anticancer therapies with precise targeting would be of significant benefit to patients with TNBC. This study used differential expression analysis based on the TCGA and GEO databases to reveal that SDC1 is significantly highly expressed in TNBC tissues and is significantly positively associated with poor patient prognosis. Further, by GO and KEGG enrichment analyses, we concentrated on deciphering the FGF family's mechanistic roles., a family of signaling proteins that have been found to be widely expressed in multicellular organisms. This family consists of at least 23 members, the function of which is to regulate cell proliferation and differentiation as well as tissue regeneration, which includes neovascularization. This process often occurs through binding to the FGF receptor at the surface of the cell membrane (FGFR), activating downstream pathways such as MAPK and PI3K [[54], [55], [56], [57]]. Furthermore, Gene Ontology (GO) analysis indicated the potential presence of SDC1 in vesicles known as "exosomes," which are nanosized extracellular vesicles secreted by cells. These vesicles are released by a diverse range of cell types and play a critical role in intercellular communication [58]. Consequently, a fundamental premise is that SDC1 orchestrates intercellular dialog through exosome-mediated paracrine networks. In summary, converging data support SDC1 as a putative mediator of exosome-driven intercellular communication between fibroblasts and stromal cells within the TME.

SDC1 is a unique biomolecule that exists in transmembrane and soluble forms. Each form plays a distinct role in tumour progression. Research indicates that membrane-bound SDC1 primarily functions as a co-receptor, interacting with extracellular matrix components via its extracellular domain to maintain cellular structural integrity and signal transduction [59,60]. In contrast, soluble SDC1 is released into the extracellular environment through the proteolytic cleavage of membrane-bound SDC1 by proteases, such as matrix metalloproteinases (MMPs) [61], a process known as 'shedding'. Our bioinformatics analysis revealed that SDC1 is significantly overexpressed in triple-negative breast cancer (TNBC) and is strongly associated with poor patient prognosis. On the tumour cell surface, transmembrane SDC1 functions as a receptor capable of directly regulating downstream signalling pathways, including the PI3K-Akt and Rap1 pathways identified in this study [30]. Concurrently, drug sensitivity analysis revealed a positive correlation between SDC1 overexpression and etoposide resistance, while molecular docking further suggested a potential direct interaction between SDC1 and etoposide. As a cell surface receptor, transmembrane SDC1 may directly participate in regulating drug uptake or controlling efflux pumps. This hypothesis was preliminarily validated by a CETSA, which demonstrated that etoposide enhances SDC1 thermal stability. While recognising the critical role of soluble SDC1 in tumour metastasis, this study focuses on the novel functions of transmembrane SDC1 in TNBC chemotherapy response and microenvironment regulation. This strategy elucidates the multifaceted role of SDC1 in TNBC and provides a theoretical basis for developing targeted therapies against transmembrane SDC1.

Senescence, which carries a certain risk of tumorigenesis, can play a pro-tumorigenic role by affecting the metabolic reprogramming of the organism. Furthermore, the process of senescence can promote tumorigenesis by affecting cellular structure and function [62,63]. Conversely, in the context of the immune system, senescence has been shown to promote tumorigenesis. The immune system, therefore, functions as both a target of senescence and a regulator of the senescence process [64]. The data presented herein indicates that SDC1 exerts a significant influence on the infiltration of immune cells within the TNBC microenvironment, with the most pronounced inhibition observed in the infiltration of CD8^+^ T cells [65], CD4^+^ T cells [66], and Macrophage M1 cells [67,68], which are recognized as the primary tumoricidal cells. Conversely, the infiltration of pro-tumorigenic immune cells, such as Macrophage M2 [69] and Mast cells [70,71], exhibited a promoting trend. Consequently, we hypothesize that further elucidation of the molecular mechanism of SDC1 and related immune cells will have an important theoretical way for targeting SDC1 in the treatment of TNBC.

Prior research has established that fibroblasts critically modulate the tumor immune milieu [72] and regulate angiogenic processes, which in turn promotes tumor progression [73]. Macrophages have also been shown to promote angiogenesis in tumors [74] and to have an important role in tumor immunity [75]. On the other hand, CAFs-derived exosome-mediated communication between CAFs and cancer cells, as well as other cells in the TME, has been demonstrated [76]. Interactions have also been observed between fibroblasts and macrophages [77]. Cells interact with each other through different signaling pathways, and we noted that fibroblasts interconnect with other cells through SDC1 regulation of related signaling pathways. In the aforementioned GO analysis, it was mentioned that SDC1 may be present in exosomes; therefore, it is reasonable to hypothesize that fibroblasts may interconnect with other cells in the tumor microenvironment through the presence of exosome SDC1. A bioinformatics analysis of SDC1's significant pro-cancer effects was conducted, and based on the findings, an *in situ* self-reactive gold nanocluster SDC1 shRNA-targeted nucleic acid delivery system was successfully constructed. This system significantly inhibited TNBC neovascularization. This bioinformatics-driven gene-targeted gold nanocluster delivery system, integrating genomic targeting and payload release, realizes the precision treatment of cancer. This investigation establishes a critical conceptual framework for optimizing therapeutic strategies against TNBC.

## 5 Conclusion

Within the present framework, we systematically revealed the biological function and clinical significance of SDC1 in TNBC by integrating multi-omics data analysis and in vitro experimental validation. The analysis of differential expression, based on data from the TCGA and GEO databases, revealed that SDC1 was significantly highly expressed in TNBC tissues and was closely associated with the poor prognosis of patients (OS, HR = 2.21, 95 % CI 1.1–4.44, *p* < 0.05; RFS, HR = 1.6, 95 % CI 1.1–2.31, *p* < 0.05; DMFS, HR = 1.63, 95 % CI 1.05–2.31, *p* < 0.05). Single-cell transcriptome sequencing revealed that SDC1 was specifically highly expressed in CAFs. Furthermore, pseudo-temporal trajectory analysis revealed that the dynamics of SDC1 expression may regulate fibroblast development. Furthermore, functional enrichment analysis indicated that SDC1 was significantly enriched in pathways related to angiogenesis, including the PI3K-Akt pathway and the Rap-1 signaling pathway. Furthermore, cellular communication and GO enrichment analyses indicated that SDC1 may play a role in the establishment of a tumor-promoting microenvironment through an exosome-mediated paracrine mechanism. Furthermore, immune infiltration analysis indicated that high SDC1 expression was significantly associated with CD8^+^ T cell and macrophage infiltration. Furthermore, Dose-response analyses demonstrated a significant positive association (r = 0.38, *p* = 0.015) linking elevated SDC1 expression with increased etoposide half-maximal inhibitory concentrations, suggesting a potential involvement of SDC1 in the mechanism of chemotherapy resistance. In consideration of the aforementioned findings, this study successfully constructed an *in situ* fluorescent self-assembled SDC1 nucleic acid delivery system. In vitro experiments confirmed the ability of this system to effectively inhibit tumor angiogenesis. This study not only elucidates the molecular mechanism by which SDC1 promotes tumor progression through multifaceted regulation of the TNBC microenvironment, providing a theoretical foundation and potential therapeutic targets for precision treatment strategies, but more significantly, leverages SDC1-focused bioinformatic analyses to construct gold nanocluster-based SDC1-shRNA delivery systems. This targeted approach effectively suppressed the pro-oncogenic functions of SDC1, demonstrating its therapeutic potential.

## CRediT authorship contribution statement

**Can Jiang:** Writing – original draft, Conceptualization. **Haixuan Wen:** Methodology, Software. **Jiabin Chen:** Resources, Software. **Na Han:** Project administration, Data curation. **Xin Sun:** Software, Methodology. **Yuzhu Zhang:** Investigation, Data curation. **Yongbin Hu:** Writing – review & editing. **Guang Shu:** Validation, Software. **Gang Yin:** Validation, Software. **Maonan Wang:** Writing – review & editing, Visualization, Supervision.

## Ethics approval and consent to participate

Not applicable.

## Consent for publication

Not applicable.

## Availability of data and materials

All datasets analyzed in this study are available for download from the TCGA database (https://portal.gdc. cancer.gov/) and the GEO database (https://www.ncbi.nlm.nih.gov/geo/).

## Funding

Not applicable.

## Declaration of competing interest

The authors declare that they have no known competing financial interests or personal relationships that could have appeared to influence the work reported in this paper.

## Figures and Tables

**Table:** 

Abbreviations	Full name
TNBC	Triple-negative breast cancer
TCGA	The Cancer Genome Atlas
GEO	Gene Expression Omnibus
OS	Overall survival
RFS	Recurrence-Free Survival
DMFS	Distant Metastasis Free Survival
HR	Hazard Ratio
CAFs	Cancer-associated fibroblasts
GO	Gene Ontology
KEGG	Kyoto Encyclopedia of Genes and Genomes
ECM	Extracellular matrix
TEM	Transmission electron microscope
HRTEM	High Resolution Transmission Electron Micro -scope
HUVECs	Human Umbilical Vein Endothelial Cells
EDS	Energy-Dispersive X-ray Spectroscopy
DEGs	Differentially expressed genes
PPI	Protein-Protein Interaction Networks
BP	Biological process
MF	Molecular function
CC	Cellular component
FGF	Fibroblast growth factor
FGFR	Fibroblast growth factor receptor
Cor	correlation coefficient
UMAP	Uniform Manifold Approximation and Projection
TME	Tumor micro-environment
GNCs	Gold nanoclusters
FTIR	Fourier transform infrared spectroscopy
SERS	Surface enhanced Raman scattering
CETSA	cell thermal shift assay

## Data Availability

Data will be made available on request.
