# Supplementary material for: Bioinformatics driven in gene targeting platform for gold anticancer strategy delivery
Source: Mater Today Bio. 2025 Oct 18;35:102438. doi: 10.1016/j.mtbio.2025.102438 (PMC12589921; doi:10.1016/j.mtbio.2025.102438)
Supplement: Multimedia component 1 [file mmc1.docx]

**Supplementary Material**

**Bioinformatics driven in gene targeting platform for gold anticancer**

**Strategy delivery**

Can Jiang^1^, Haixuan Wen^1^, Jiabin Chen^1^, Na Han^1^, Xin Sun^1^, Yuzhu Zhang^1^, Yongbin Hu^1^, Guang Shu^1^, Gang Yin^1^, Maonan Wang^1*^

^1^Department of Pathology, Xiangya hospital, Xiangya School of Basic Medical Sciences, Central South University, Changsha, China

^*^Corresponding author, email: maonanwang@csu.edu.cn

**Supplementary Figures**


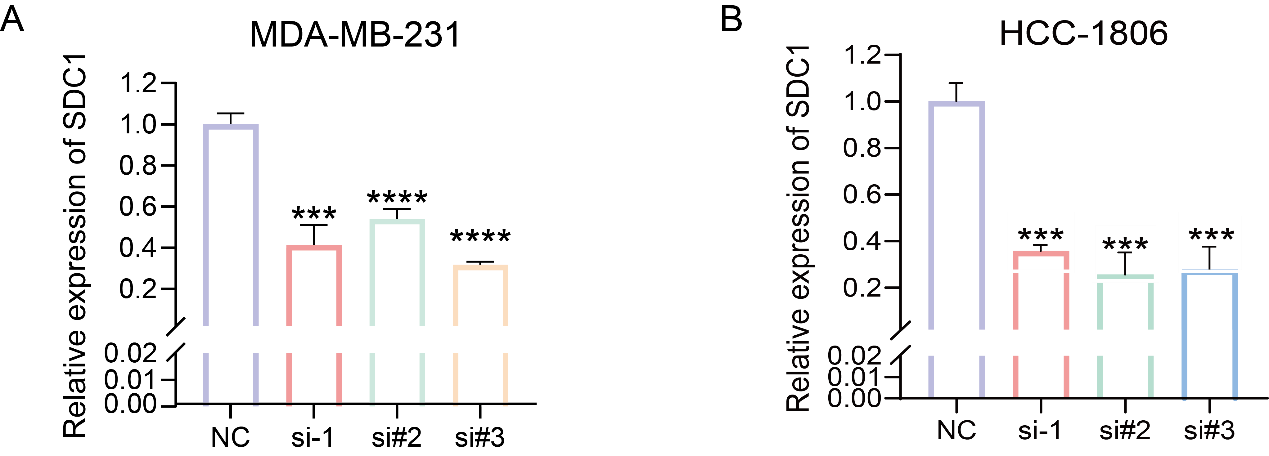


Figure S1. Knockdown efficiency of SDC1 by different siRNA sequences in

MDA-MB-231 (A) and HCC-1806 (B).*p < 0.05, **p < 0.01, ***p < 0.001, ****p < 0.0001


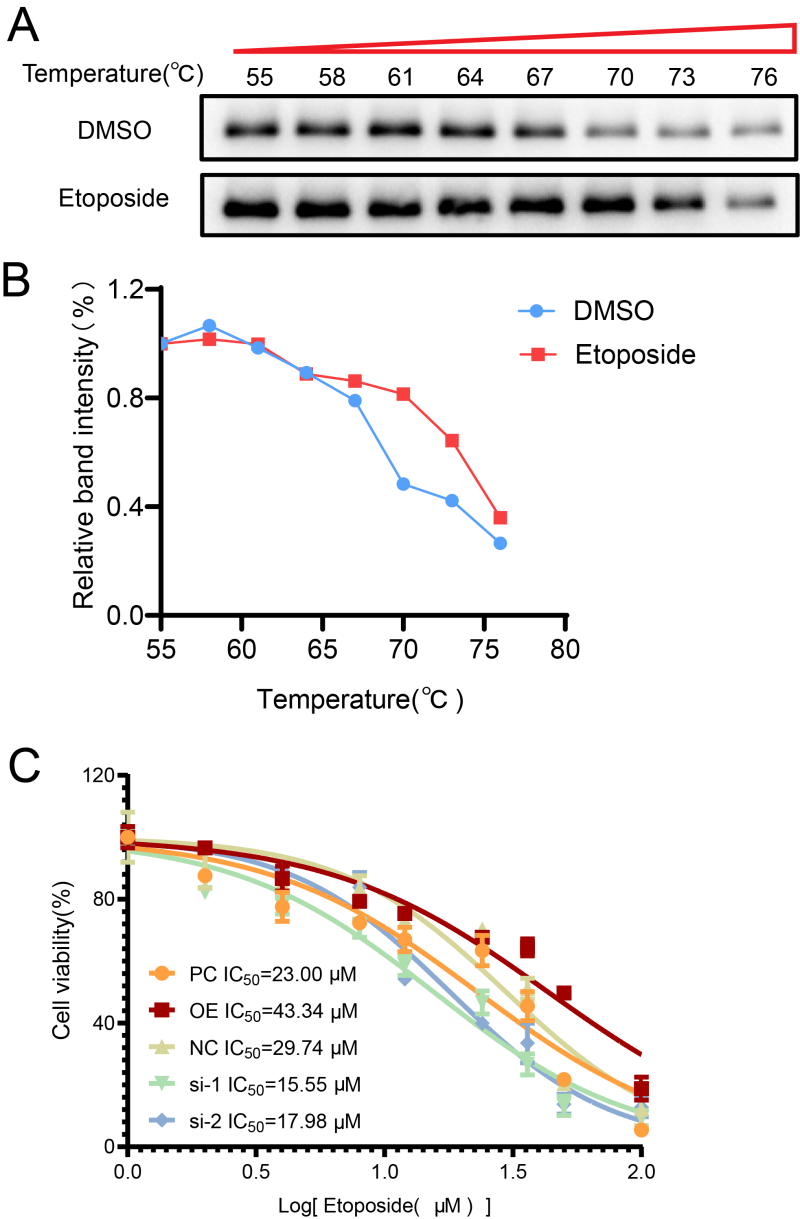


Figure S2. Thermal stability of SDC1 and changes in etoposide IC_50._ **(A)** Protein expression of SDC1 in CETSA; **(B)** Protein expression of SDC1; **(C)**Variations in the IC50 of etoposide.


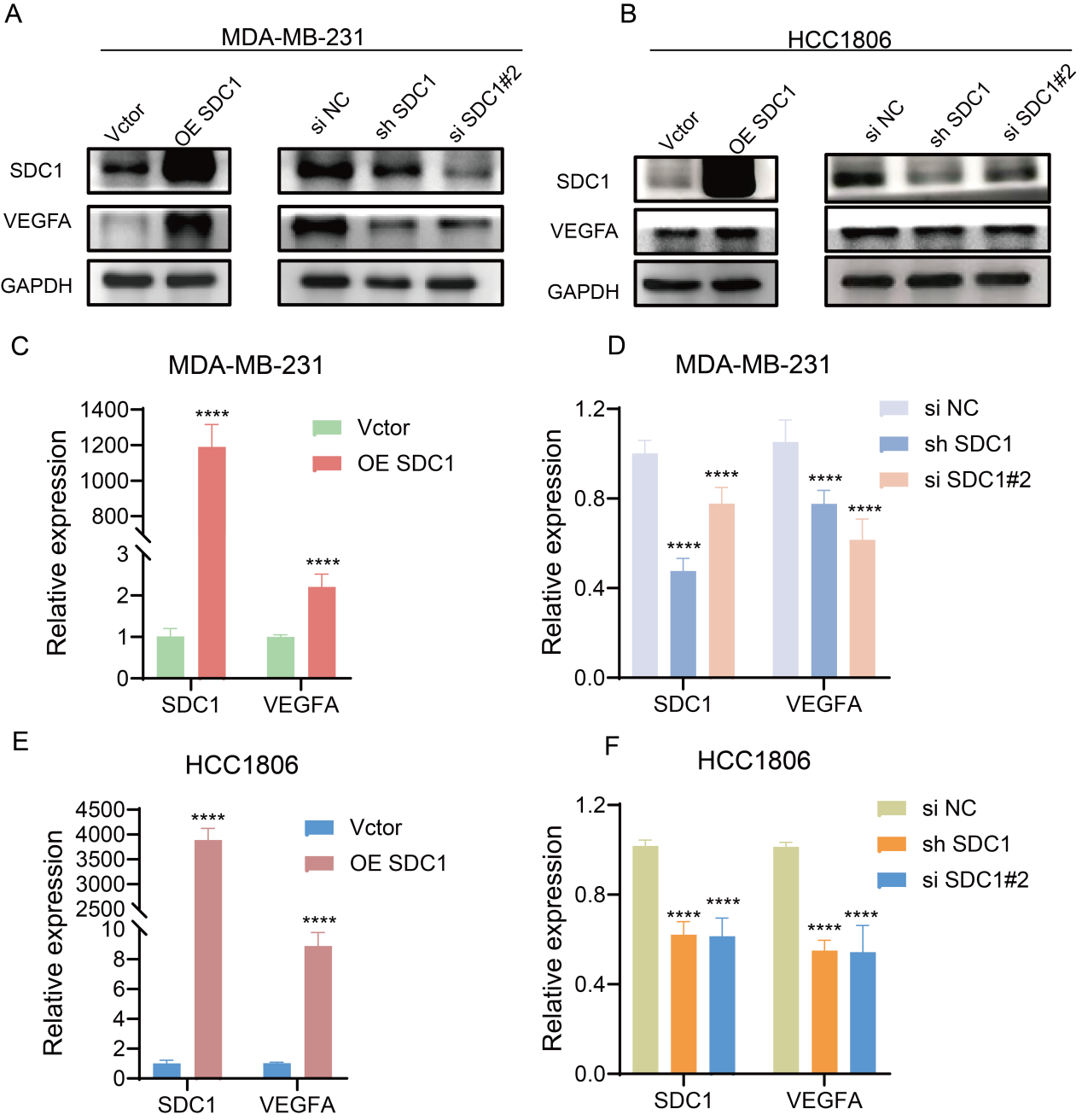


Figure s3. Expression of SDC1 and VEGFA at mRNA and protein levels. **(A)** Levels of protein expression of SDC1 and VEGFA in MDA-MB-231 cells；**（B）**Levels of protein expression of SDC1 and VEGFA in HCC1806 cells. **（C-D）**The level of expression of SDC1 and VEGFA as mRNA in MDA-MB-231 cells；**（E-F）**The level of expression of SDC1 and VEGFA as mRNA in HCC1806 cells.*p < 0.05, **p < 0.01, ***p < 0.001, ****p < 0.0001


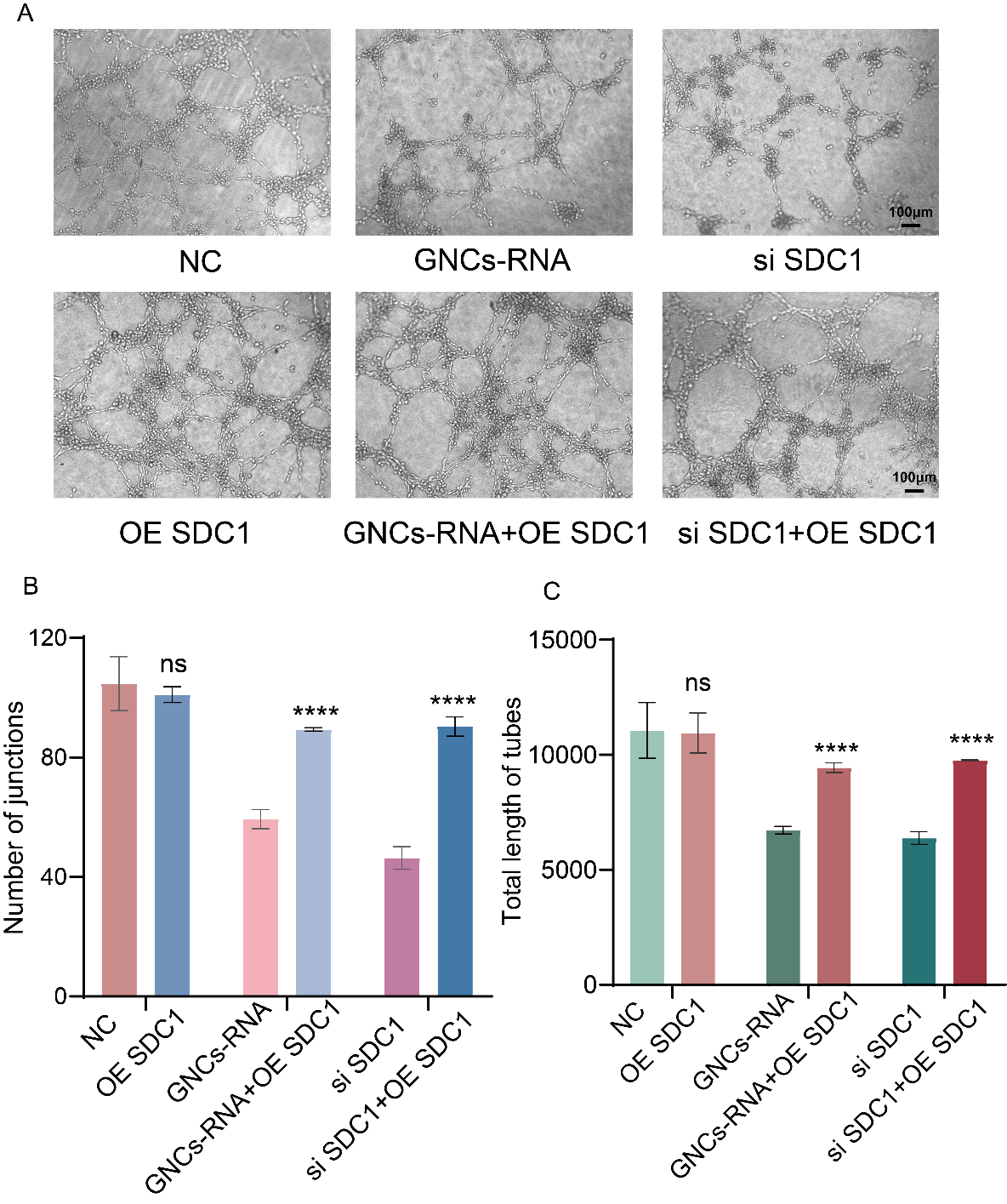


Figure S4. The rescue of *in vitro* tube formation assay. (A)Images of SDC1 re-expression rescue in an *in vitro* tube formation assay. (B-C) Analysis of angiogenic capacity. The number and total length of tube connections indicate the angiogenic capacity of each group. Magnification 100X, scale bar 100 μm. *p < 0.05, **p < 0.01, ***p < 0.001, ****p < 0.0001


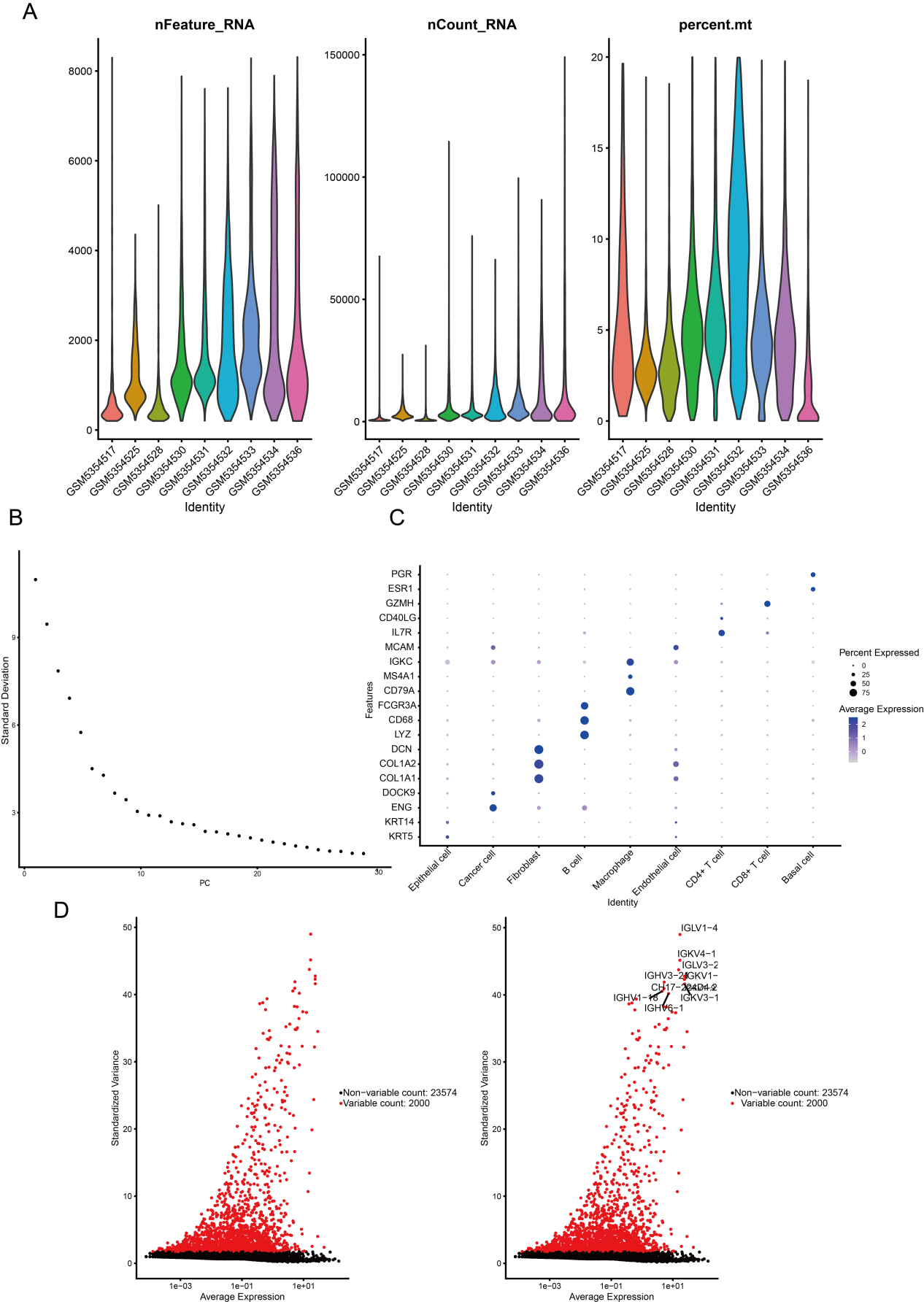

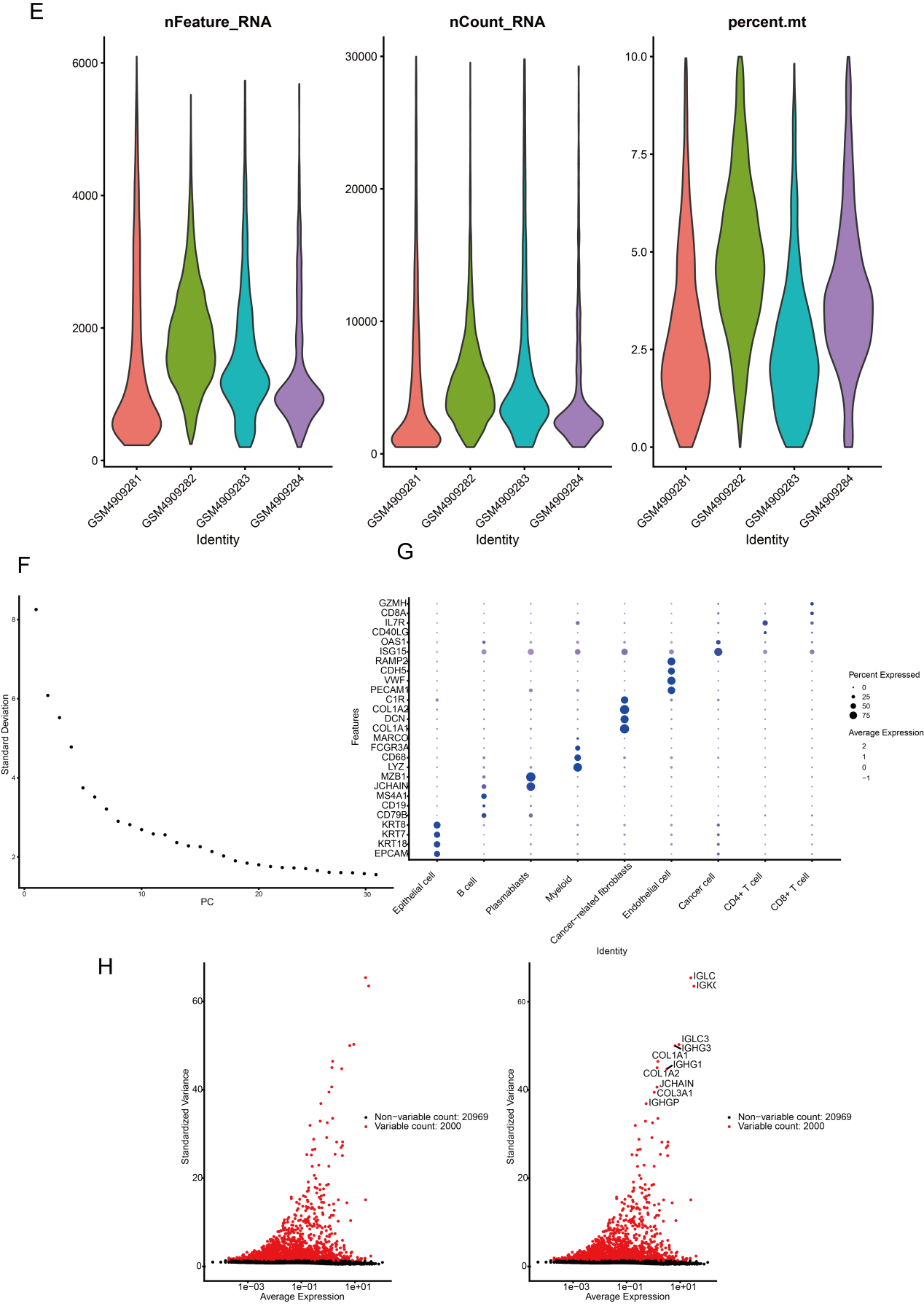


Figure S5. Visualization of single-cell data processing. **(A, E)** Visualization after data filtering. **(B, F)** Dimensionality reduction effects, with the first 30 dimensions used for downstream analysis. **(C, G)** Key marker genes for each cell cluster. **(D, H)** Display of top 10 highly variable gene names.

**Supplementary table**

Table 1. The corresponding peak assignment of SERS spectra

| Peaks | Assignment |
| --- | --- |
| 645 cm^-1^ | C-C twisting mode of phenylalanine |
| 679 cm^-1^ | Ring breathing modes in the DNA bases |
| 746 cm^-1^ | T |
| 1002 cm^-1^ | C-C aromatic ring stretching |
| 1127 cm^-1^ | C-N |
| 1243 cm^-1^ | PO_2_^-^ |
| 1309 cm^-1^ | CH_3_/CH_2_ twisting |
| 1337 cm^-1^ | A,G |
| 1447 cm^-1^ | CH_2_ bending model |
| 1585 cm^-1^ | C=C oleﬁnic stretch |
| 1667 cm^-1^ | C=C stretching band |

**Supplementary Methods**

**Western blot analysis**

Total protein was extracted from cell samples using RIPA lysis buffer (Beyotime, Shanghai, China) supplemented with a protease inhibitor cocktail. Total proteins were separated by SDS-PAGE electrophoresis and transferred to PVDF membranes (Cytiva, USA) for detection. Subsequently, membranes were blocked in TBST solution containing 5% skim milk for 2 hours,

incubated with primary antibody overnight at 4°C, and incubated with secondary antibody for 1 hour at room temperature. Final visualization was achieved using chemiluminescent substrate (Biosharp, Anhui, China). The specific primary antibodies were as follows: SDC1( Proteintech 10593-1-AP, 1:3000 )GAPDH (Utibody; UM4002,1:3000), VEGFA (LITHO; LTO0784, 1:3000).

**Cell Thermal Shift Assay (CETSA)**

For CETSA in cell lysates, MDA-MB-231 cells were harvested and subjected to three freeze-thaw cycles using liquid nitrogen. Lysates were diluted with 2 mL buffer and divided into two groups: one treated with etoposide and the other serving as a control (DMSO). After incubation at room temperature for 2 hours, transfer 100 μL of cell lysate from each group into new Eppendorf tubes (8 tubes per group). Heat the lysates at 55°C, 58°C, 62°C, 64°C, 67°C, 70°C, 73°C, and 76°C in a PCR machine (BioRed), then cool for 3 minutes. The lysates were subsequently analyzed by Western blotting.
